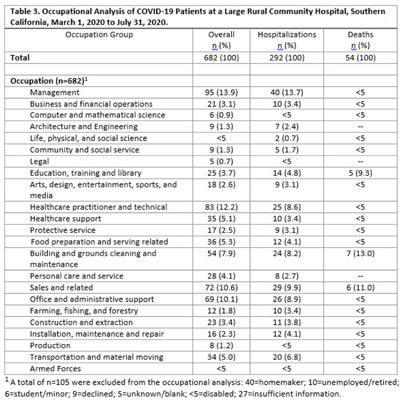# Epidemiologic risk factors and occupation analysis of COVID-19 cases, hospitalizations, and deaths–southern California, 2020

**DOI:** 10.1017/ash.2022.119

**Published:** 2022-05-16

**Authors:** Patricia Cummings, Theresa Ubano Perez, Megan Sidana, Charmaine Peters

## Abstract

**Background:** COVID-19 occupational exposures have been examined using death certificates and employment data from the Bureau of Labor Statistics and the O*Net database in the United States. However, no studies have examined cases, hospitalizations, and deaths by occupation using hospital records.^1^ We analyzed COVID-19 cases using hospitalization data from a large, rural community hospital to fill this gap in the evidence base. **Methods:** A retrospective cross-sectional study design was used to examine patients with COVID-19 from March 1 through July 31, 2020. We examined demographic characteristics, such as age, sex, race or ethnicity, and length of stay (LOS), among those who tested positive for SARS-CoV-2. Epidemiological risk factors were also analyzed, including smoking status, body mass index (BMI), alcohol use, and occupation. Occupational data were processed using the National Institute for Occupational Safety and Health Industry and Occupation Computerized Coding System. Homemakers, disabled persons or retirees, students or minors, and listed occupations with insufficient information were excluded from the analysis. Occupations were categorized into 23 major occupation groups based on the 2018 Standard Occupational Classification System. To examine whether certain occupations are at a higher risk due to COVID-19, we stratified the analysis by overall cases, hospitalizations, and deaths. Microsoft Power BI Desktop and IBM SPSS version 28.0.0.0 software were used to analyze the data. This study was reviewed and approved by the local institutional review board. **Results:** In total, 2,132 COVID-19 diagnoses with 1,049 total hospitalizations were identified during the study period. Most cases were in the group aged 50–64 years, white race, and/or Hispanic ethnicity (Table 1). Most cases never or rarely drank alcohol, were nonsmokers, and had a BMI ≥30 (Table 2). The average LOS among those hospitalized for COVID-19 was 6.46 days. The occupational analysis revealed a higher frequency of cases among those in management (n = 95, 14%) and healthcare (n = 83, 12%), with those in management (n = 40, 14%) and sales (n = 29, 10%) having the highest frequency of being hospitalized. However, the highest frequency of deaths occurred among those in building and grounds cleaning and maintenance occupations (13%) (Table 3). **Conclusions:** This study describes the burden of COVID-19 in a rural area with a large aging population and highlights potential health disparities among severe cases and deaths in different occupational groups.

1. Baker MG, Peckham TK, Seixas NS. Estimating the burden of United States workers exposed to infection or disease: a key factor in containing risk of COVID-19 infection. *PLoS One* 2020;15:e0232452.

**Funding:** None

**Disclosures:** None

**Table:**
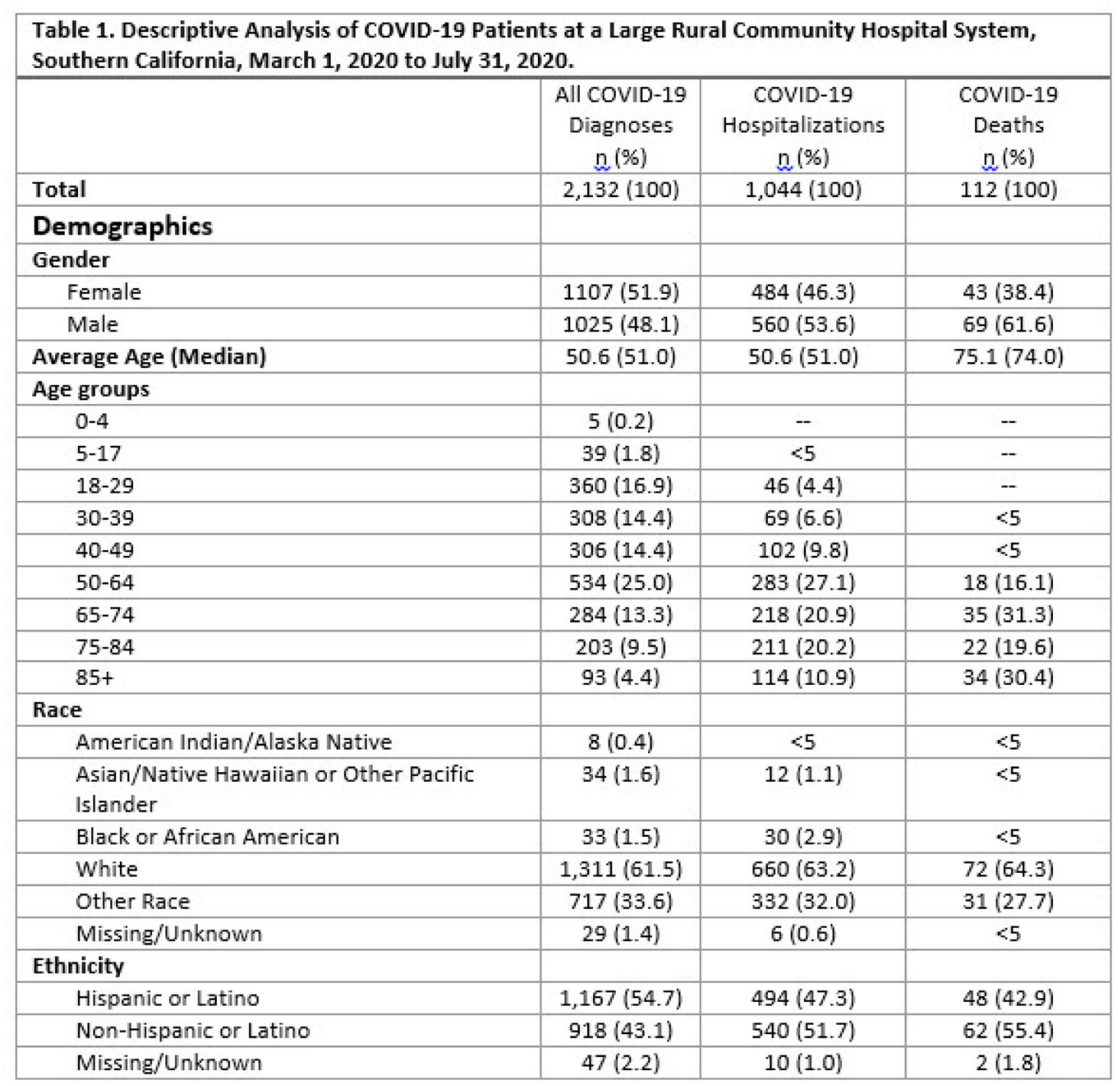

**Table:**
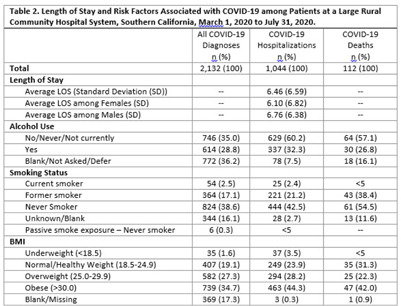

**Table:**